# An Observational Laboratory-Based Assessment of SARS-CoV-2 Molecular Diagnostics in Benin, Western Africa

**DOI:** 10.1128/mSphere.00979-20

**Published:** 2021-01-13

**Authors:** Anna-Lena Sander, Anges Yadouleton, Andres Moreira-Soto, Carine Tchibozo, Gildas Hounkanrin, Yvette Badou, Carlo Fischer, Nina Krause, Petas Akogbeto, Edmilson F. de Oliveira Filho, Anges Dossou, Sebastian Brünink, Christian Drosten, Melchior A. Joël Aïssi, Mamoudou Harouna Djingarey, Benjamin Hounkpatin, Michael Nagel, Jan Felix Drexler

**Affiliations:** a Charité-Universitätsmedizin Berlin, corporate member of Freie Universität Berlin, Humboldt-Universität zu Berlin, and Berlin Institute of Health, Berlin, Germany; b Laboratoire des Fièvres Hémorragiques Virales du Benin, Cotonou, Benin; c Ecole Normale Supérieure de Natitingou, Université Nationale des Sciences, Technologies, Ingénierie et Mathématiques (UNSTIM), Natitingou, Benin; d Ministry of Health, Cotonou, Benin; e Conseil National de Lutte contre le VIH-Sida, la Tuberculose, le Paludisme, les IST et les Epidémies, Cotonou, Benin; f World Health Organization, Regional Office for Africa, Health Emergencies Programme, Brazzaville, Congo; g Deutsche Gesellschaft für Internationale Zusammenarbeit (GIZ) GmbH, Bonn, Germany; h German Centre for Infection Research (DZIF), associated partner Charité-Universitätsmedizin Berlin, Berlin, Germany; U.S. Centers for Disease Control and Prevention

**Keywords:** coronavirus, SARS-CoV-2, COVID-19, Benin, West Africa, RT-PCR

## Abstract

Information on severe acute respiratory syndrome coronavirus 2 (SARS-CoV-2) spread in Africa is limited by insufficient diagnostic capacity. Here, we assessed the coronavirus disease (COVID-19)-related diagnostic workload during the onset of the pandemic in the central laboratory of Benin, Western Africa; characterized 12 SARS-CoV-2 genomes from returning travelers; and validated the Da An RT-PCR-based diagnostic kit that is widely used across Africa. We found a 15-fold increase in the monthly laboratory workload due to COVID-19, dealt with at the cost of routine activities. Genomic surveillance showed near-simultaneous introduction of distinct SARS-CoV-2 lineages termed A.4 and B.1, including the D614G spike protein variant potentially associated with higher transmissibility from travelers from six different European and African countries during March-April 2020. We decoded the target regions within the ORF1ab and N genes of the Da An dual-target kit by MinION-based amplicon sequencing. Despite relatively high similarity between SARS-CoV-2 and endemic human coronaviruses (HCoVs) within the ORF1ab target domain, no cross-detection of high-titered cell culture supernatants of HCoVs was observed, suggesting high analytical specificity. The Da An kit was highly sensitive, detecting 3.2 to 9.0 copies of target-specific *in vitro* transcripts/reaction. Although discrepant test results were observed in low-titered clinical samples, clinical sensitivity of the Da An kit was at least comparable to that of commercial kits from affluent settings. In sum, virologic diagnostics are achievable in a resource-limited setting, but unprecedented pressure resulting from COVID-19-related diagnostics requires rapid and sustainable support of national and supranational stakeholders addressing limited laboratory capacity.

**IMPORTANCE** Months after the start of the COVID-19 pandemic, case numbers from Africa are surprisingly low, potentially because the number of SARS-CoV-2 tests performed in Africa is lower than in other regions. Here, we show an overload of COVID-19-related diagnostics in the central laboratory of Benin, Western Africa, with a stagnating average number of positive samples irrespective of daily sample counts. SARS-CoV-2 genomic surveillance confirmed a high genomic diversity in Benin introduced by travelers returning from Europe and other African countries, including early circulation of the D614G spike mutation associated with potentially higher transmissibility. We validated a widely used RT-PCR kit donated by the Chinese Jack Ma Foundation and confirmed high analytical specificity and clinical sensitivity equivalent to tests used in affluent settings. Our assessment shows that although achievable in an African setting, the burden from COVID-19-related diagnostics on national reference laboratories is very high.

## Abstract (precis)

Months after the start of the COVID-19 pandemic, case numbers from Africa are surprisingly low, potentially because the number of SARS-CoV-2 tests performed in Africa is lower than in other regions. Here, we show an overload of COVID-19-related diagnostics in the central laboratory of Benin, Western Africa, with a stagnating average number of positive samples irrespective of daily sample counts.

## OBSERVATION

**FIG 1:**
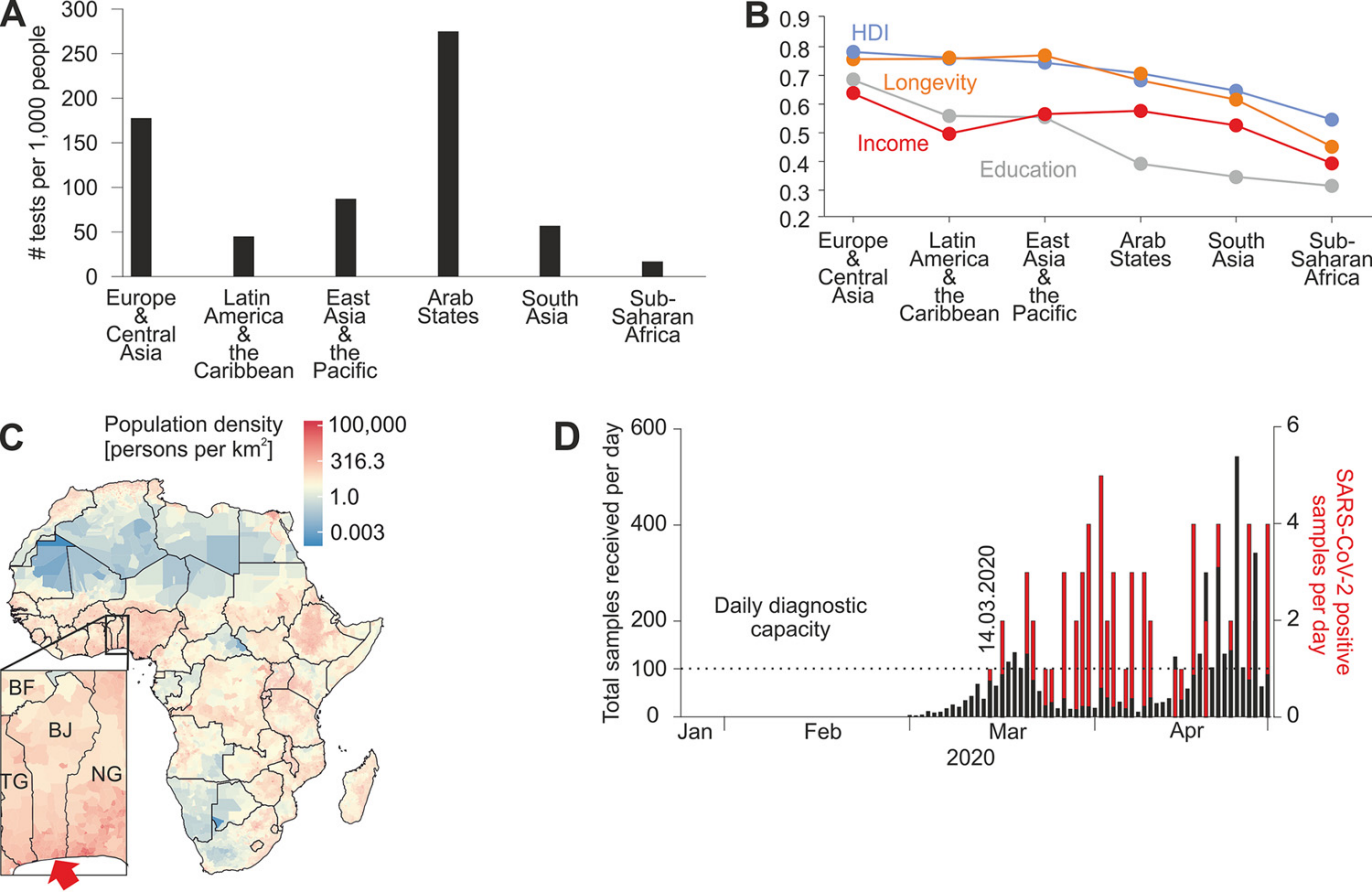
Africa’s development status and COVID-19 workload at the reference laboratory of Benin. (A) Total number of SARS-CoV-2 tests performed relative to the size of population, received as freely available data from https://ourworldindata.org/coronavirus-testing#the-scale-of-testing-compared-to-the-scale-of-the-outbreak (accessed 14 September 2020). The newest entry for each country was selected, and countries were allocated to defined regions. Bars represent mean tests performed for the region. (B) Regional human development indices. Longevity (orange), income (red), and education (gray) indices and the human development index (HDI) (blue) as the geometric mean of the three aforementioned indices are shown. Data were obtained as freely available data from http://hdr.undp.org/en/data, accessed 15 June 2020. (C) Population density map constructed using freely available data from https://www.worldpop.org/doi/10.5258/SOTON/WP00004 (accessed 9 June 2020). Benin (BJ) is shown below. BF, Burkina Faso; TG, Togo; NG, Nigeria. The red arrow denotes the location of the Laboratoire des Fièvres Hémorragiques Virales du Benin (LFHB) in Cotonou. (D) COVID-19 workload at the LFHB. Overall SARS-CoV-2 daily diagnostic requests received at the LFHB until 28 April 2020 (black) and positive cases confirmed per day at the LFHB (red). Dotted lines denote the range of maximal daily diagnostic capacity of LFHB. Marked is 14 March 2020, the day of the first confirmed SARS-CoV-2 case in Benin.

The coronavirus disease (COVID-19) pandemic causes excessive pressure on health care systems particularly in resource-limited settings like Africa (1). Surprisingly, several months after the start of the pandemic, case numbers and deaths reported from Africa remain low compared to other regions, potentially due to nonpharmaceutical interventions and the relatively young African population potentially associated with milder courses of COVID-19 (2). However, lack of diagnostic capacity is likely a major factor additionally limiting surveillance in Africa (3), since sub-Saharan countries, one of the most underdeveloped regions globally, perform considerably less SARS-CoV-2 testing than in affluent settings (Fig. 1A and B).

Benin is a West African country with 12 million inhabitants located in one of the most populous regions of sub-Saharan Africa (Fig. 1C). As of mid-September, Benin reported only 2,267 cases and 40 deaths from COVID-19 (4). The Laboratoire des Fièvres Hémorragiques Virales du Benin (LFHB) is Benin’s central laboratory for respiratory diseases. Specificity of commercially available antibody enzyme-linked immunosorbent assays (ELISAs) validated for use in affluent settings can be limited in Africa, highlighting the importance of careful validation of diagnostic tests on site (5).

We assessed the setup of SARS-CoV-2 molecular diagnostics at LFHB during the onset of the pandemic. Oronasopharyngeal swabs for SARS-CoV-2 diagnostics were first received 1 March, and the first case was detected 14 March 2020. Until 28 April 2020, LFHB had received a total of 4,382 samples for SARS-CoV-2 molecular testing with up to 543 samples per day (Fig. 1D). LFHB can process up to about 100 samples daily due to the limited availability of personnel, reagents, and laboratory equipment. This threshold was exceeded on 14 days during March-April, demonstrating the immense workload that LFHB has compensated for using night shifts and its entire workforce for SARS-CoV-2 diagnostics, at the cost of viral hemorrhagic fever surveillance. The average number of positive samples per day was 1.4 (range 1 to 5), irrespective of the number of samples received, suggesting an imprecise country-level test strategy.

**FIG 2:**
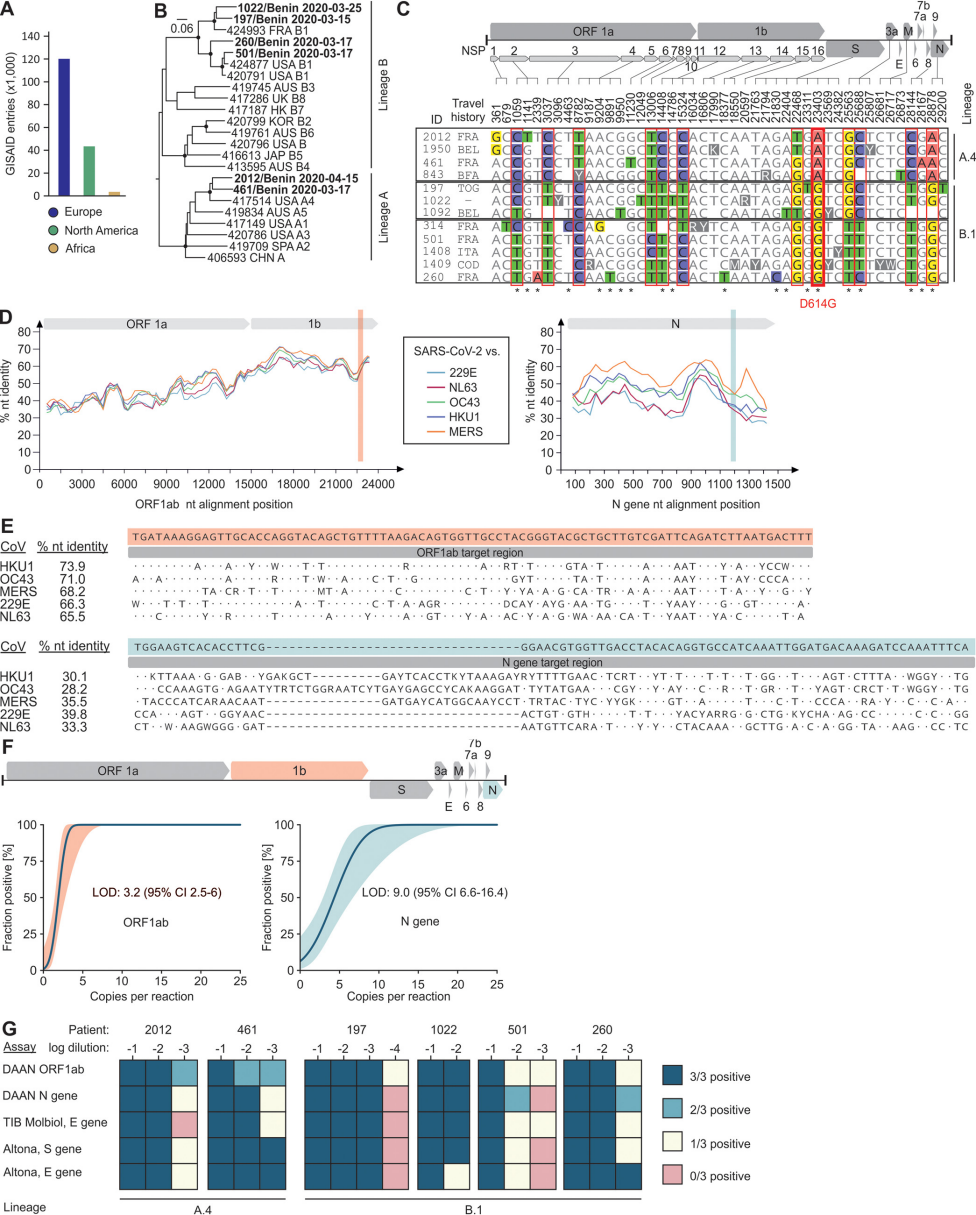
Phylogenetic analyses of SARS-CoV-2 in Africa and molecular diagnostic test validation. (A) SARS-CoV-2 sequence entries from Africa and affluent settings in GISAID on 10 November 2020. (B) Phylogenetic tree inferred using BEAST2 showing 23 complete SARS-CoV-2 genomes globally sampled from humans. Posterior support is shown for nodes >0.90 as filled circles. Benin-derived sequences are shown in bold. Only sequences without missing information were used for this analysis. Sequences are designated with GISAID accession IDs/country of origin/lineage according to reference 7. (C) Alignment showing all variable sites across Benin derived SARS-CoV-2 genomes from this study. Empty spaces indicate lack of sequence information. Red boxes denote variable nucleotide positions in at least three or more sequences. Gray boxes denote groups belonging to lineage A.4 or B.1 according to the work of Rambaut et al. (7). Asterisks indicate nonsynonymous substitutions. Nucleotide positions correspond to the SARS-CoV-2 reference genome (GenBank accession number MN908947). BEL, Belgium; BFA, Burkina Faso; COD, Democratic Republic of the Congo; FRA, France; ITA, Italy; TOG, Togo; NSP, nonstructural protein; ORF, open reading frame. (D) Nucleotide (nt) identity plot within the ORF1ab (left) and N (right) genes between SARS-CoV-2 and endemic HCoVs. Top, schematic representation of the ORF1ab and N gene. Highlighted are the locations of the ORF1ab and N gene target regions of the Da An kit. (E) Alignments showing the ORF1ab (top) and N (bottom) gene target regions of SARS-CoV-2 and endemic HCoVs. Shown are the 100% consensus sequences of all sequences in the final data sets of each HCoV. nt, nucleotide. (F) Analytical sensitivity of the Da An real-time RT-PCR kit for both assays using target region-specific IVTs. The solid line shows predicted proportion of positive results at a given input; colored lines show the 95% CI. The genome illustration highlights the target genes of the assays. CI, confidence interval; LOD, lower limit of detection. (G) SARS-CoV-2 assay comparison using clinical samples.

Genomic sequencing of SARS-CoV-2 is important to elucidate transmission chains and to identify mutations potentially changing the viral phenotype (6). By 10 November 2020, 195,771 SARS-CoV-2 fully or partially sequenced genomes had been deposited in GISAID by the global scientific community. While affluent countries published more than 83% of those sequences, only 1.8% (*n* = 3,580) originated from Africa (Fig. 2A). To investigate the SARS-CoV-2 diversity in Benin, we determined 12 SARS-CoV-2 genomes from Beninese citizens. Full genomes were determined for six of those strains, and nearly complete genomes were determined for another six strains (88 to 98% completeness). Only one of these 12 individuals was a patient from a local hospital, whereas 11 were returning travelers from European or Central-West African countries who underwent mandatory SARS-CoV-2 testing upon entering Benin (Table 1). A full-genome-based phylogeny revealed the occurrence of both globally circulating lineages A and B in those travelers (Fig. 2B) (7).

**TABLE 1:** Characteristics of SARS-CoV-2-positive patients from which SARS-CoV-2 full genomes were generated

Sample ID	Male/female	Age (yr)	Location	Travel history	Symptoms	Sampling date (day.mo.yr)	*C_T_* value^*a*^
197	M	34	Oueme	None	None	15.03.2020	28.2
260	F	29	Cotonou	France	None	17.03.2020	30.1
314	F	42	Cotonou	France	None	17.03.2020	35.9
461	F	45	Cotonou	France	None	17.03.2020	31.0
501	F	61	Cotonou	France	None	17.03.2020	32.7
843	F	41	Natitingou	Burkina Faso	None	19.03.2020	31.8
1022	F	29	Cotonou	Togo	None	25.03.2020	32.9
1092	M	38	Cotonou	Belgium	None	27.03.2020	37.0
1408	M	39	Cotonou	Italy	None	04.04.2020	34.1
1409	M	44	Cotonou	DR Congo	None	06.04.2020	34.7
1950	M	52	Cotonou	Belgium	Fever	15.04.2020	33.5
2012	M	34	Cotonou	France	None	15.04.2020	30.8

a *C_T_*, threshold cycle; tested with a real-time-RT SARS-CoV-2 E-gene assay described in https://www.who.int/docs/default-source/coronaviruse/protocol-v2-1.pdf.

To identify variation within those two lineages, we analyzed nucleotide differences within the 12 Benin-derived sequences (8). In total, 12 variable nucleotide positions were observed, resulting in seven amino acid exchanges known from other strains globally (Fig. 2C) (9). Four Benin-derived strains belonged to lineage A.4 reported predominantly from the United States until mid-September 2020. Since none of those travelers returned from the United States, this finding highlights the global dispersion of SARS-CoV-2 variants and likely sample biases in the existing data sets (Table 1). Another eight strains belonged to lineage B.1, a globally circulating lineage originating in Europe (7), which was consistent with the individual travel histories (Table 1). Sampling of those divergent SARS-CoV-2 strains from travelers within only 4 weeks is consistent with global mixing of SARS-CoV-2 variants early during the pandemic and with recent sequence reports from South Africa, Kenya, and Nigeria (10–12). Of note, all eight strains belonging to lineage B.1 harbored a nucleotide exchange resulting in the D614G spike protein variant, which may be associated with increased transmissibility (13). Whether this variant has since become the predominant lineage circulating in Benin requires further investigation. In sum, genomic analyses suggest multiple introductions of at least two globally circulating SARS-CoV-2 lineages as well as the D614G mutation into Africa before mid-April 2020, highlighting the value of closing borders to decrease virus importation into Benin and other African countries.

Access to state-of-the-art reagents for diagnostics of acute SARS-CoV-2 infections is limited in resource-limited regions (14). To address this, the Chinese Jack Ma Foundation donated over 1 million RT-PCR-based SARS-CoV-2 kits produced by the Chinese Da An Gene Corporation to all African countries in March 2020 (15). While the SARS-CoV-2 target genes ORF1ab and N are informed by the kit’s manufacturer, the exact target sites remained unknown, hindering further analyses of assay performance.

We decoded the target sites of both assays included in the Da An kit using MinION-based amplicon sequencing. Although SARS-CoV-2 differs substantially from endemic human coronaviruses (HCoVs) in the ORF1ab and N genes (Fig. 2D), cross-detection of endemic HCoVs may be possible in the ORF1ab-based assay due to relatively high nucleotide identity of 66 to 74% between SARS-CoV-2 and HCoVs (Fig. 2E), which is consistent with preliminary data on potentially unspecific results of the Da An kit (16). However, testing of high-titered HCoV cell culture supernatants revealed no cross-detection in either of the assays, suggesting limited risks of false-positive test results. Nevertheless, exhaustive testing of clinical specimens from Africa representing diverse pathogens will be required to allow definite conclusions.

Preliminary validation suggested high sensitivity of the Da An kit, but the exact limits of detection remained unclear (16). Testing of assay-specific *in vitro* transcripts (IVTs) revealed high analytical sensitivity for both assays at 3.2 (ORF1ab) and 9.0 (N) copies/reaction (Fig. 2F). Using endpoint dilutions of six clinical samples, we observed discrepant results between the two Da An assays at lower SARS-CoV-2 concentrations (Fig. 2G). The discrepant results suggested that caution must be taken upon validating tests of patient specimens containing low amounts of viral RNA, such as those sampled late during the course of infection (17).

Nucleotide mutations in binding regions of PCR oligonucleotides are known to affect the diagnostic sensitivity of SARS-CoV-2 assays, potentially leading to false-negative results (18). Although the exact oligonucleotide binding sites of the Da An assays remained unknown, it should be noted that the Da An kit detected SARS-CoV-2 RNA in clinical samples covering both the SARS-CoV-2 genetic lineages A.4 and B.1 identically or superior to kits from affluent settings (TIB Molbiol and Altona GmBH, Germany) (Fig. 2G). Database comparison revealed nucleotide exchanges within the ORF1ab and N gene target regions in less than 2% of >100,000 SARS-CoV-2 genomic sequences available in public databases (see Fig. S1 and S2 in the supplemental material). The limited variation within globally circulating variants within the Da An kit target domains thus suggests robust detection of diverse SARS-CoV-2 sublineages to date.

10.1128/mSphere.00979-20.1 FIG S1 Variability of SARS-CoV-2 sequences within the ORF1ab target domain of the Da An kit. The figure shows all 114 unique sequences differing from the majority consensus. Sequence accession numbers as well as numbers of identical sequences for each unique sequence are listed in Data Set S1. Download FIG S1, TIF file, 1.9 MB. Copyright © 2021 Sander et al. 2021 Sander et al. This content is distributed under the terms of the Creative Commons Attribution 4.0 International license.

10.1128/mSphere.00979-20.2 FIG S2 Variability of SARS-CoV-2 sequences within the N gene target domain of the Da An kit. The figure shows all 154 unique sequences differing from the majority consensus. Sequence accession numbers as well as numbers of identical sequences for each unique sequence are listed in Data Set S2. Download FIG S2, TIF file, 2.1 MB. Copyright © 2021 Sander et al. 2021 Sander et al. This content is distributed under the terms of the Creative Commons Attribution 4.0 International license.

Our data highlight that despite many challenges, appropriate SARS-CoV-2 testing can be achieved in an African setting to inform public health control measures (19). Although our study is limited by the small number of samples used for RT-PCR validation and genomic surveillance, consistent analytical and clinical sensitivity and similarly high SARS-CoV-2 genomic diversity in neighboring countries suggest robustness of our data.

Philanthropic efforts to provide test kits and to enhance pathogen genomic sequencing (20) are important steps to support surveillance and patient care in Africa, but sustainable action will require improvement of supply chains and laboratory infrastructure. The advances in access to HIV diagnostics and therapeutics in Africa demonstrate that such goals are ambitious but not unachievable (21). Strengthening of national and supranational stakeholders coordinating the continent’s response to COVID-19 is needed to accelerate evidence-based public health responses (22).

### Study design and participants.

We assessed daily data on sample receipt and RT-PCR-based SARS-CoV-2 detections during January to April 2020. We characterized SARS-CoV-2 from 12 PCR-confirmed travelers entering Benin during March-April 2020 (Table 1). This study was approved by the ethics committee of the Ministry of Health (Arrêté 2020 no. 030/MS/DC/SGM/DNSP/CJ/SA/027SGG2020) and followed the Declaration of Helsinki. Written consent was obtained from all patients participating in the study. Anonymized data sets were used, and all analysis of personally identifiable data took place only in the LFHB.

### Laboratory testing.

RNA was extracted from oronasopharyngeal swabs suspended in 140 μl of viral transport medium using the viral RNA minikit (Qiagen, Germany) following the manufacturer’s instructions. Diagnostics of SARS-CoV-2 was performed using the kit donated by the Jack Ma Foundation termed 2019 Novel Coronavirus RNA detection kit (Da An Gene Co., Ltd., of Sun Yat-sen University, China), which targets the ORF1ab and N genes of SARS-CoV-2, and the RealStar SARS-CoV-2 RT-PCR kit 1.0 (Altona Diagnostics, Germany), which targets the E and S genes of SARS-CoV-2, as well as the SarbecoV E-gene kit and SARS-CoV-2 RdRP kit (TIB Molbiol, Germany). For testing analytical sensitivity of the ORF1ab and N gene assays of the Da An kit, we designed assay-specific IVTs for the respective genomic target regions based on the SARS-CoV-2 strain 2019-nCoV/München-1.2/2020/985 (EPI ISL 406862). Target regions of both Da An assays were decoded by amplicon sequencing using the MinION platform from Oxford Nanopore Technologies using the ligation kit LSK-109 and the expansion kit EXP-BC096 according to the manufacturer’s instructions. Periamplicon regions were amplified using suitable Artic primers (nCoV-2019_69 and nCoV-2019_96; LEFT and RIGHT) available on GitHub (https://github.com/artic-network/artic-ncov2019/tree/master/primer_schemes/nCoV-2019/V3) and cloned and *in vitro* transcribed as previously described (23). Photometric IVT quantification was done as previously described (24). Probit regression analyses to determine the lower limit of detection of real-time RT-PCR assays were performed as described earlier (25) using dilutions of quantified IVT RNA standards. Limit-of-detection (LOD) analyses were done using SPSS V22 (IBM, Germany) using eight parallel test replicates. Cell culture supernatants containing endemic human coronaviruses used for specificity testing were quantified using strain-specific real-time RT-PCRs and IVT quantification standards generated as described above (copies/µl: HCoV-HKU1, 1.5 × 10^3^; HCoV-OC43, 9.0 × 10^5^; HCoV-NL63, 3.7 × 10^6^; HCoV-229E, 3.0 × 10^5^, Middle East respiratory syndrome coronavirus [MERS-CoV], 3.3 × 10^5^). All samples were tested with the Da An kit in four technical replicates.

Whole-genome amplification was done using the Artic Consortium PCR-based protocol (https://artic.network/ncov-2019). Library preparation and Illumina MiSeq sequencing were done using the KAPA Frag kit and KAPA Hyper Prep kit (Roche Molecular Diagnostics, Switzerland) and MiSeq reagent v2 chemistry (Illumina, USA) according to the manufacturers’ protocols. Genome assembly was done by mapping MiSeq reads to a representative African SARS-CoV-2 sequence (HCoV-19/Senegal/611/2020|EPI_ISL_420076). Genome annotations were made in analogy to a SARS-CoV-2 reference sequence (NC045512) using Geneious 9.1.8 (Biomatters, Auckland, New Zealand).

### *In silico* analyses.

Time-stamped Bayesian phylogeny based on sampling dates was performed in BEAST2 (https://www.beast2.org/). A codon position-specific general time-reversible (GTR) substitution model with γ-distributed rates among sites was used. SARS-CoV-2 MT019529 from Wuhan, China, was used as an outgroup. The majority consensus of 10,000 trees from the posterior distribution with mean branch lengths was sampled. Subsequently, the phylogeny was annotated with TreeAnnotator and visualized in FigTree from the BEAST package (https://beast.community/programs).

Similarity plots between SARS-CoV-2 and endemic HCoVs were calculated in SSE version 1.3 (http://www.virus-evolution.org/Downloads/Software) using a global deletion option and a sliding window of 1,000 and a step size of 250 nucleotides for ORF1ab and a sliding window of 160 and a step size of 40 nucleotides for the N gene. Coding sequences of ORF1ab and N genes were extracted and translationally aligned using Geneious 9.1.8. Alignments included one representative sequence of each CoV (SARS-CoV-2 [MN908947], HCoV-229E [NC002645], HCoV-NL63 [AY567487], HCoV-OC43 [AY585228], HCoV-HKU1 [NC006577], and MERS-CoV [JX869059]).

Sequence comparison of the ORF1ab and N gene Da An kit target regions between SARS-CoV-2 and endemic HCoVs was performed as follows. BLAST searches using the ORF1ab or N gene target sequences of the respective CoV were performed on 9 November 2020. Duplicates, cell culture-adapted strains, or viruses isolated from experimentally infected animals were excluded. Remaining sequences of each data set were translationally aligned. The final data sets included HKU1 (*n* = 39), OC43 (*n* = 178), MERS (*n* = 584), 229E (*n* = 34), and NL63 (*n* = 85) sequences for ORF1ab and HKU1 (*n* = 66), OC43 (*n* = 239), MERS (*n* = 592), 229E (*n* = 80), and NL63 (*n* = 107) sequences for the N gene. Nucleotide positions of target regions in HCoVs as listed earlier were as follows: HKU1, 21075 to 21162; OC43, 20818 to 20905; MERS, 20824 to 20911; 229E, 19884 to 19971; and NL63, 19791 to 19878 (ORF1ab), and HKU1, 29305 to 29403; OC43, 30070 to 30177; MERS, 29506 to 29598; 229E, 26614 to 26688; NL63, 27016 to 27090 (N). SARS-CoV-2 nucleotide positions of the ORF1ab and N gene target regions were 20880 to 20967 and 29238 to 29312, respectively.

SARS-CoV-2 nucleotide variability within the ORF1ab and N gene target domains of the Da An kit was analyzed as follows. All available SARS-CoV-2 sequences on GISAID were downloaded on 3 November 2020. Sequences that were nonhuman, duplicates, shorter than 29,700 nucleotides, or containing more than 1% N bases were removed, resulting in a final data set of 101,851 SARS-CoV-2 sequences. The data set was aligned using MAFFT, and the ORF1ab and N gene target regions of all sequences were extracted. One hundred twenty-five and 105 sequences containing N bases indicating poor sequence quality within this region were removed for the ORF1ab and N gene target region, respectively. 101,048 and 100,057 sequences showed no variation in the ORF1ab or N gene target domain, respectively, whereas 678 and 1,689 sequences showed nucleotide exchanges within the ORF1ab and N gene target domain, respectively.

### Data availability.

All full genomes generated within this study were deposited to GISAID (IDs EPI_ISL_476822 to EPI_ISL_476831 and EPI_ISL_476833 to EPI_ISL_476834).

10.1128/mSphere.00979-20.3 DATA SET S1 List of unique sequences shown in Fig. S1. The sequence name shows the GISAID accession number, origin, sample date, and number of identical sequences within the ORF1ab target region in public databases. Sequences without GISAID accession numbers are available at https://civnb.info/sequences/. Download Data Set S1, PDF file, 2.1 MB. Copyright © 2021 Sander et al. 2021 Sander et al. This content is distributed under the terms of the Creative Commons Attribution 4.0 International license.

10.1128/mSphere.00979-20.4 DATA SET S2 List of unique sequences shown in Fig. S2. The sequence name shows the GISAID accession number, origin, sample date, and number of identical sequences within the N gene target region in public databases. Sequences without GISAID accession numbers are available at https://civnb.info/sequences/. Download Data Set S2, PDF file, 2.1 MB. Copyright © 2021 Sander et al. 2021 Sander et al. This content is distributed under the terms of the Creative Commons Attribution 4.0 International license.
