# Supplementary figures and images for: An Observational Laboratory-Based Assessment of SARS-CoV-2 Molecular Diagnostics in Benin, Western Africa
Source: mSphere. 2021 Jan 13;6(1):e00979-20. doi: 10.1128/mSphere.00979-20 (PMC7845609; doi:10.1128/mSphere.00979-20)

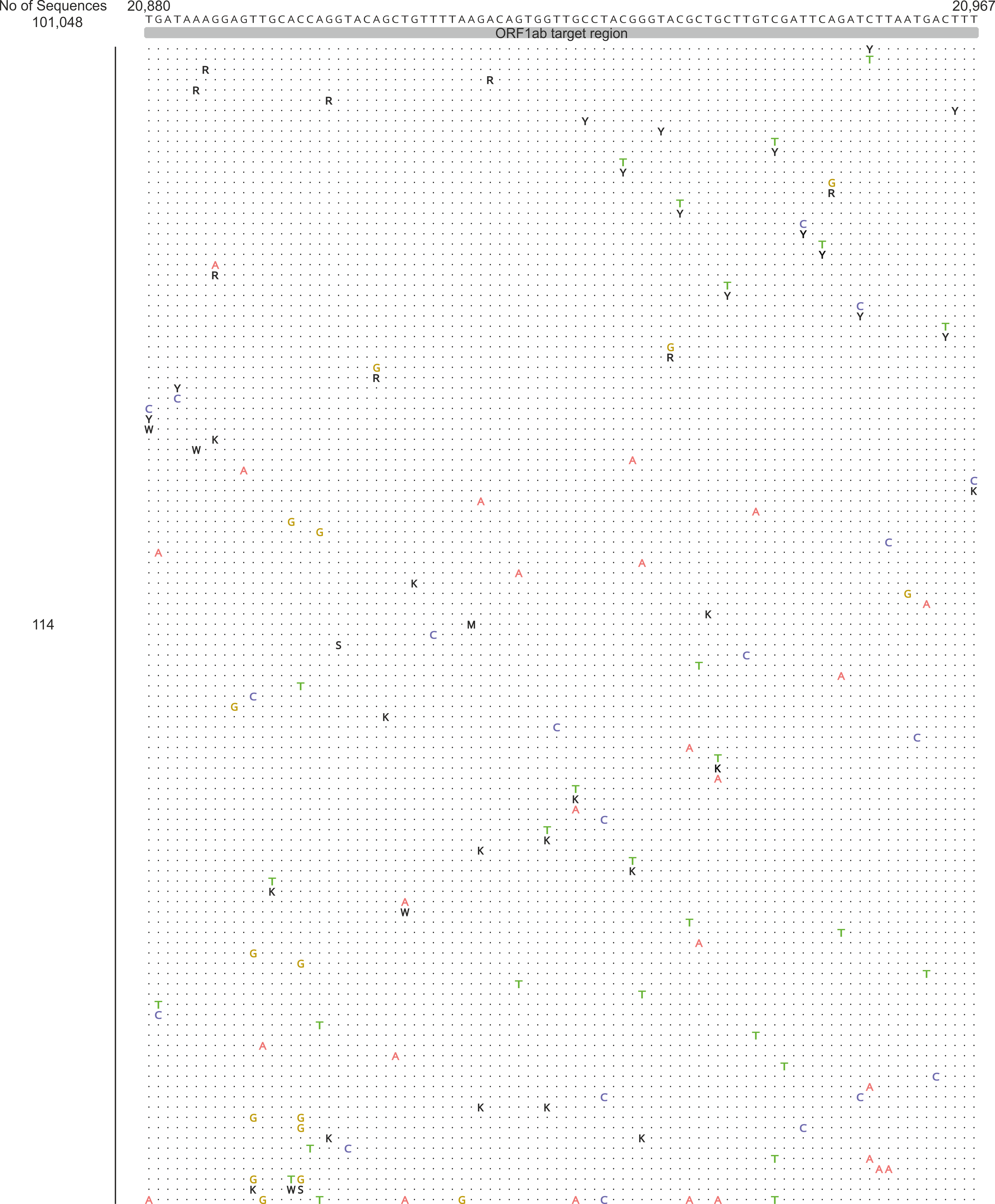

Supplement: FIG S1 [file mSphere.00979-20-sf001.tif]

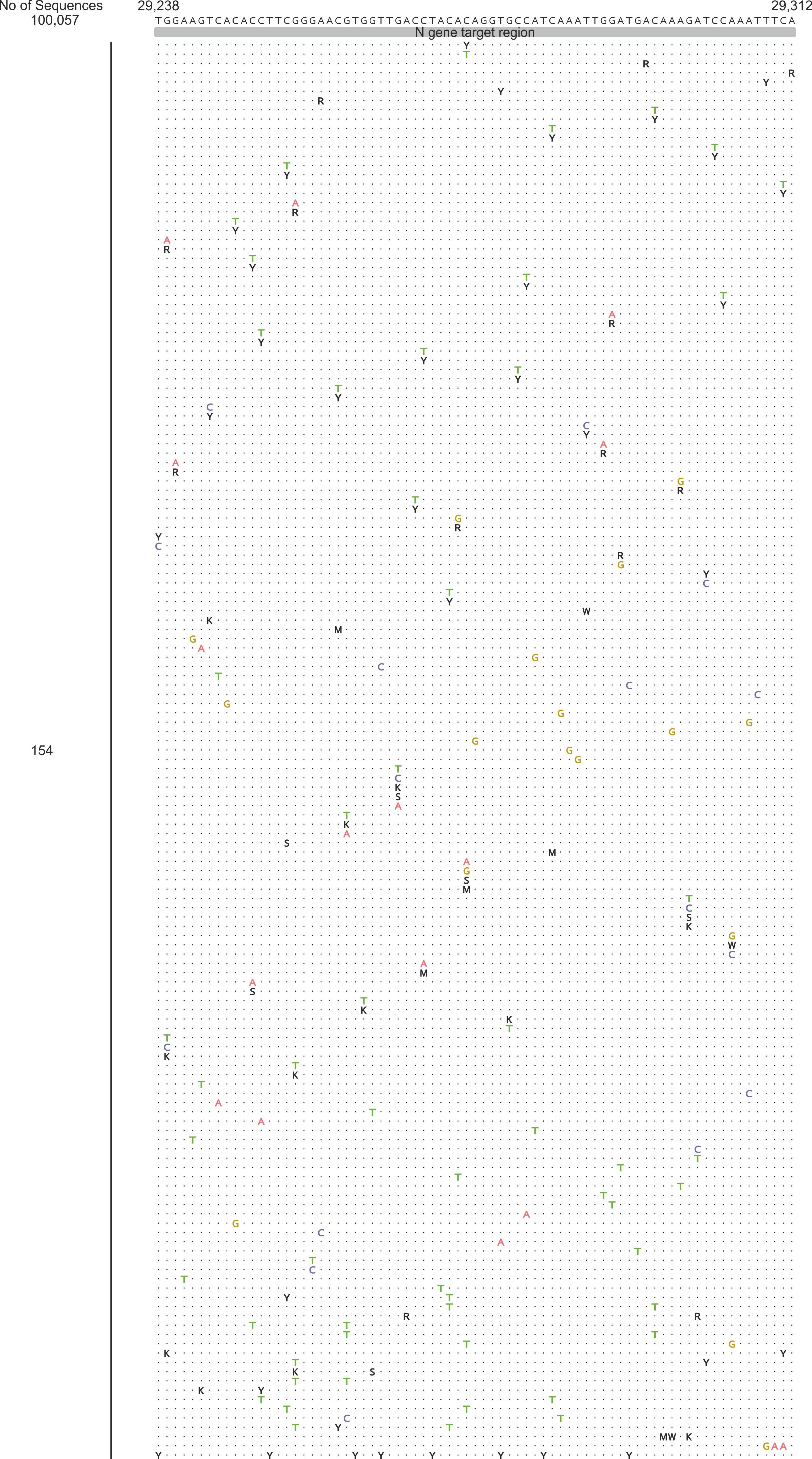

Supplement: FIG S2 [file mSphere.00979-20-sf002.tif]
