# Supplementary material for: An Observational Laboratory-Based Assessment of SARS-CoV-2 Molecular Diagnostics in Benin, Western Africa
Source: mSphere. 2021 Jan 13;6(1):e00979-20. doi: 10.1128/mSphere.00979-20 (PMC7845609; doi:10.1128/mSphere.00979-20)
Supplement: DATA SET S1 [file mSphere.00979-20-sd001.pdf]

Supplementary dataset 1

EPI_ISL_402125|Asia-China-unknown-unknown|2019-12-31 (101048)

EPI_ISL_441673|Europe-United_Kingdom-England-unknown|2020-04-06 (7)

EPI_ISL_420365|Europe-Belgium-Liege-unknown|2020-03-19 (9)

EPI_ISL_486467|Europe-Switzerland-Valais-unknown|2020-04-01 (3)

EPI_ISL_426828|Oceania-Australia-Victoria-unknown|2020-03-25 (2)

EPI_ISL_589472|Europe-United_Kingdom-England-unknown|2020-09-24 (2)

EPI_ISL_565289|Oceania-Australia-Victoria-unknown|2020-07-23

EPI_ISL_565155|Oceania-Australia-Victoria-unknown|2020-07-27

EPI_ISL_510716|Europe-Switzerland-Zurich-unknown|2020-07-14

EPI_ISL_442610|Europe-United_Kingdom-England-unknown|2020-04-07

BetaCoV/Blaubeuren/MD570082171176/2020 (147)

EPI_ISL_556755|Europe-United_Kingdom-England-unknown|2020-07-16 (5)

BetaCoV/Ploen/ChVir8321/2020 (39)

EPI_ISL_451702|Europe-Switzerland-Bern-unknown|2020-03-16 (2)

EPI_ISL_557762|Europe-United_Kingdom-England-unknown|2020-06-29 (28)

EPI_ISL_452374|Europe-Spain-Andalucia-Velez_Malaga|2020-03-15

EPI_ISL_411929|Asia-South_Korea-unknown-unknown|2020-01-XX (27)

EPI_ISL_593501|North_America-Canada-Ontario-Ottawa|2020-05-13

EPI_ISL_541691|Asia-India-Maharashtra-unknown|2020-07-18 (24)

EPI_ISL_527378|Asia-Singapore-unknown-unknown|2020-08-25

EPI_ISL_435360|North_America-USA-Utah-unknown|2020-04-03 (14)

EPI_ISL_589441|Europe-United_Kingdom-England-unknown|2020-05-05 (3)

EPI_ISL_416503|Europe-France-Bretagne-Rennes|2020-03-01 (12)

EPI_ISL_419756|Oceania-Australia-Victoria-unknown|2020-03-11

EPI_ISL_432276|Europe-United_Kingdom-Wales-unknown|2020-04-07 (11)

EPI_ISL_566696|Europe-United_Kingdom-England-unknown|2020-09-10 (3)

EPI_ISL_498274|Asia-Bangladesh-Chittagong-Brahmanbaria|2020-07-19 (5)

EPI_ISL_469100|Asia-Singapore-unknown-unknown|2020-03-26

EPI_ISL_455112|Europe-Netherlands-Flevoland-unknown|2020-03-31 (5)

EPI_ISL_599095|Europe-United_Kingdom-Scotland-unknown|2020-10-01

EPI_ISL_525915|North_America-USA-Oregon-Washington_County|2020-06-29 (4)

EPI_ISL_436433|Asia-India-Delhi-unknown|2020-04-05

EPI_ISL_483914|Europe-United_Kingdom-England-unknown|2020-06-23 (2)

EPI_ISL_476131|Europe-Switzerland-Basel-Landschaft-unknown|2020-04-19

EPI_ISL_430691|Oceania-Australia-Victoria-unknown|2020-04-11

EPI_ISL_609413|Europe-United_Kingdom-England-unknown|2020-10-06

EPI_ISL_532156|Europe-United_Kingdom-England-unknown|2020-05-11

EPI_ISL_475997|Asia-Singapore-unknown-unknown|2020-01-31

EPI_ISL_445344|South_America-Chile-Temuco-unknown|2020-03-26

EPI_ISL_526759|Europe-United_Kingdom-England-unknown|2020-04-22

EPI_ISL_486178|North_America-USA-California-Orange_County|2020-04-28

EPI_ISL_462002|Europe-United_Kingdom-England-unknown|2020-05-22 (25)

EPI_ISL_436928|North_America-USA-Utah-unknown|2020-04-15 (4)

EPI_ISL_484860|North_America-USA-Wisconsin-Jackson_County|2020-06-01 (2)

EPI_ISL_514080|North_America-USA-unknown-unknown|2020-04-22

EPI_ISL_438460|Europe-United_Kingdom-England-unknown|2020-03-30 (2)

EPI_ISL_440584|Europe-United_Kingdom-England-unknown|2020-04-02 (2)

EPI_ISL_602389|Europe-Russia-Leningrad-Kirishi|2020-09-18 (2)

EPI_ISL_445182|North_America-USA-California-San_Francisco_County|2020-04-12 (2)

EPI_ISL_507007|Asia-Iran-Tehran-unknown|2020-05-01 (2)

EPI_ISL_524756|Asia-India-Gujarat-Patan|2020-07-06 (2)

EPI_ISL_545719|North_America-USA-Houston-unknown|2020-07-06 (2)

EPI_ISL_471243|North_America-USA-Wisconsin-unknown|2020-04-03 (2)

EPI_ISL_451819|Europe-Switzerland-Basel-Landschaft-unknown|2020-03-31 (2)

EPI_ISL_526520|Europe-United_Kingdom-Scotland-unknown|2020-08-10

EPI_ISL_447389|Asia-Israel-North_District-unknown|2020-03-29

EPI_ISL_603679|Europe-Switzerland-Graubunden-unknown|2020-10-14

EPI_ISL_562167|Oceania-Australia-Victoria-unknown|2020-03-27

EPI_ISL_601149|Europe-United_Kingdom-Scotland-unknown|2020-09-22

EPI_ISL_451790|Europe-Switzerland-Basel-Landschaft-unknown|2020-03-25

EPI_ISL_556849|Europe-United_Kingdom-England-unknown|2020-07-24

EPI_ISL_571390|North_America-USA-Nevada-unknown|2020-03-18

EPI_ISL_454227|Europe-Portugal-unknown-unknown|2020-03-17

BetaCoV/Berlin/ChVir4453-colon/2020

EPI_ISL_458001|North_America-USA-North_Carolina-unknown|2020-04-XX

EPI_ISL_594570|Europe-United_Kingdom-England-unknown|2020-10-05

EPI_ISL_608871|Europe-United_Kingdom-England-unknown|2020-10-09

EPI_ISL_417538|Europe-Iceland-Reykjavik-unknown|2020-03-17

EPI_ISL_529034|North_America-USA-New_York-unknown|2020-03-08

EPI_ISL_541953|Europe-Spain-Balear_Islands-Palma_de_Mallorca|2020-04-01

BetaCoV/Gifhorn/570067165661/2020 (29)

EPI_ISL_515101|Africa-Ghana-unknown-unknown|2020-05-22 (4)

EPI_ISL_455292|Europe-Netherlands-Utrecht-unknown|2020-03-12

EPI_ISL_433827|Europe-United_Kingdom-England-unknown|2020-04-08 (2)

EPI_ISL_516571|Europe-Switzerland-Zurich-unknown|2020-08-04

EPI_ISL_550683|Europe-United_Kingdom-England-unknown|2020-08-30 (2)

EPI_ISL_441213|Europe-United_Kingdom-England-unknown|2020-04-03 (4)

EPI_ISL_475586|North_America-USA-California-Los_Angeles_County|2020-03-22 (2)

EPI_ISL_574908|Europe-Switzerland-Basel-Stadt-unknown|2020-03-17

EPI_ISL_592187|Oceania-Australia-Victoria-unknown|2020-08-06

EPI_ISL_419943|Oceania-Australia-Victoria-unknown|2020-03-21 (92)

EPI_ISL_489981|Europe-Switzerland-Zurich-unknown|2020-07-02 (4)

EPI_ISL_422017|Europe-United_Kingdom-Wales-unknown|2020-03-29 (8)

EPI_ISL_528399|Europe-United_Kingdom-England-unknown|2020-04-14 (2)

EPI_ISL_602658|Africa-South_Africa-KZN-unknown|2020-09-01 (2)

EPI_ISL_607658|Europe-United_Kingdom-England-unknown|2020-10-15

EPI_ISL_418405|Europe-Finland-unknown-unknown|2020-03-14 (11)

EPI_ISL_554814|Europe-United_Kingdom-England-unknown|2020-06-11 (2)

EPI_ISL_523097|Europe-Netherlands-Noord_Brabant-unknown|2020-05-30 (5)

EPI_ISL_416153|Europe-Denmark-Copenhagen-unknown|2020-03-02 (10)

EPI_ISL_454521|Asia-India-Maharashtra-unknown|2020-04-18 (5)

EPI_ISL_483849|Asia-India-Gujarat-Gondal|2020-06-12 (5)

EPI_ISL_525288|North_America-USA-Utah-unknown|2020-05-15 (2)

BetaCoV/Berlin/572023233/2020

EPI_ISL_454558|Asia-India-Maharashtra-unknown|2020-04-13 (2)

EPI_ISL_493084|North_America-USA-Missouri-St._Louis|2020-06-19

EPI_ISL_539716|Asia-India-Telangana-unknown|2020-08-11 (2)

EPI_ISL_463554|North_America-USA-Washington-Yakima_County|2020-04-30 (7)

EPI_ISL_461754|Europe-United_Kingdom-Scotland-unknown|2020-04-26 (6)

EPI_ISL_605283|North_America-USA-Utah-unknown|2020-07-18

EPI_ISL_542857|North_America-USA-Houston-unknown|2020-05-16

EPI_ISL_547613|North_America-USA-Wisconsin-Trempealeau_County|2020-07-14

EPI_ISL_516499|North_America-USA-Wisconsin-Dane_County|2020-07-26 (2)

EPI_ISL_510857|Europe-Sweden-Vasternorrland-unknown|2020-06-01

EPI_ISL_591886|Oceania-Australia-Victoria-unknown|2020-08-11

EPI_ISL_452348|Asia-China-Beijing-unknown|2020-03-18

EPI_ISL_577659|Asia-India-Maharashtra-unknown|2020-09-18 (2)

EPI_ISL_476119|Europe-Switzerland-Zurich-unknown|2020-04-27

EPI_ISL_452335|Asia-China-Beijing-unknown|2020-03-16 (2)

EPI_ISL_511104|Europe-Portugal-unknown-unknown|2020-05-06

EPI_ISL_534517|Europe-United_Kingdom-Scotland-unknown|2020-08-21 (2)

EPI_ISL_486401|Asia-India-Karnataka-unknown|2020-05-04

EPI_ISL_436445|Asia-India-Delhi-unknown|2020-04-09

EPI_ISL_471467|Asia-China-Guangdong-unknown|2019-XX-XX (2)
