# Supplementary material for: An Observational Laboratory-Based Assessment of SARS-CoV-2 Molecular Diagnostics in Benin, Western Africa
Source: mSphere. 2021 Jan 13;6(1):e00979-20. doi: 10.1128/mSphere.00979-20 (PMC7845609; doi:10.1128/mSphere.00979-20)
Supplement: DATA SET S2 [file mSphere.00979-20-sd002.pdf]

Supplementary dataset 2

EPI_ISL_402125|Asia-China-unknown-unknown|2019-12-31 (100057)

EPI_ISL_450452|North_America-USA-California-Santa_Clara_County|2020-03-25 (18)

EPI_ISL_421399|North_America-USA-New_York-Manhattan|2020-03-17 (117)

EPI_ISL_547629|North_America-USA-Wisconsin-La_Crosse_County|2020-07-19

EPI_ISL_475939|Asia-Singapore-unknown-unknown|2020-04-21

EPI_ISL_451838|Europe-Switzerland-Zurich-unknown|2020-03-31

EPI_ISL_556077|Europe-United_Kingdom-England-unknown|2020-06-21

EPI_ISL_443238|Asia-Singapore-unknown-unknown|2020-03-17

EPI_ISL_421343|North_America-USA-Wisconsin-Dane_County|2020-03-24 (110)

EPI_ISL_476108|Europe-Switzerland-Basel-Stadt-unknown|2020-04-30 (9)

EPI_ISL_429559|Europe-Denmark-unknown-unknown|2020-03-25 (93)

EPI_ISL_476079|Europe-Switzerland-Basel-Landschaft-unknown|2020-05-15 (7)

EPI_ISL_406533|Asia-China-Guangdong-Guangzhou|2020-01-22 (87)

EPI_ISL_516592|Europe-Switzerland-Zurich-unknown|2020-08-05 (2)

BetaCoV/Oelsnitz/572026382/2020 (68)

EPI_ISL_445353|South_America-Chile-Santiago-unknown|2020-04-02 (9)

BetaCoV/Berlin/MD570043617546/2020 (57)

EPI_ISL_438405|Europe-United_Kingdom-England-unknown|2020-03-30 (5)

BetaCoV/Berlin/ChVir8329/2020 (56)

EPI_ISL_575831|Europe-United_Kingdom-England-unknown|2020-08-19

EPI_ISL_419872|Oceania-Australia-Victoria-unknown|2020-03-18 (46)

EPI_ISL_513725|North_America-USA-California-Orange_County|2020-05-25 (2)

EPI_ISL_421598|North_America-USA-New_York-Manhattan|2020-03-21 (34)

EPI_ISL_609421|Europe-United_Kingdom-England-unknown|2020-10-07

EPI_ISL_429777|Europe-Luxembourg-unknown-unknown|2020-03-24 (31)

EPI_ISL_541958|Europe-Spain-Balear_Islands-Palma_de_Mallorca|2020-03-17 (4)

EPI_ISL_434022|Europe-United_Kingdom-England-unknown|2020-04-14 (28)

EPI_ISL_441311|Europe-United_Kingdom-England-unknown|2020-04-02 (2)

EPI_ISL_475835|Europe-Austria-unknown-unknown|2020-04-21 (27)

EPI_ISL_530538|Europe-United_Kingdom-England-unknown|2020-08-17

EPI_ISL_418985|Europe-Belgium-Ellezelles-unknown|2020-03-09 (27)

EPI_ISL_598530|Europe-United_Kingdom-England-unknown|2020-10-06

BetaCoV/Schweinfurt/572021984/2020 (17)

EPI_ISL_467266|Europe-Spain-Galicia-Santiago_de_Compostela|2020-04-15 (5)

EPI_ISL_427439|North_America-USA-Wisconsin-Rock_County|2020-04-10 (12)

EPI_ISL_465596|Europe-United_Kingdom-England-unknown|2020-05-04 (2)

EPI_ISL_445741|Europe-United_Kingdom-Wales-unknown|2020-04-12 (12)

EPI_ISL_467012|Europe-Switzerland-Basel-Stadt-unknown|2020-05-08 (2)

EPI_ISL_427111|Oceania-Australia-Victoria-unknown|2020-04-04 (10)

EPI_ISL_513716|North_America-USA-California-Orange_County|2020-05-22

EPI_ISL_420382|Europe-Belgium-Oudergem-unknown|2020-03-24 (5)

EPI_ISL_426425|North_America-USA-Maryland-unknown|2020-03-04

EPI_ISL_444371|Europe-United_Kingdom-England-unknown|2020-04-29 (4)

EPI_ISL_430623|Oceania-Australia-Victoria-unknown|2020-04-10

EPI_ISL_493543|Europe-United_Kingdom-England-unknown|2020-07-04 (4)

EPI_ISL_451786|Europe-Switzerland-Graubunden-unknown|2020-03-25 (2)

EPI_ISL_482081|Europe-United_Kingdom-England-unknown|2020-05-04 (3)

EPI_ISL_422186|Europe-United_Kingdom-Wales-unknown|2020-03-29

EPI_ISL_528493|North_America-USA-Alaska-unknown|2020-07-14 (2)

EPI_ISL_468348|North_America-USA-California-Santa_Clara_County|2020-04-25

EPI_ISL_426984|Oceania-Australia-Victoria-unknown|2020-03-27 (2)

EPI_ISL_566338|Europe-United_Kingdom-England-unknown|2020-09-16 (2)

EPI_ISL_603240|Asia-Bangladesh-Dhaka-unknown|2020-10-08

EPI_ISL_554604|Europe-United_Kingdom-England-unknown|2020-06-12

EPI_ISL_466954|Europe-Switzerland-Basel-Stadt-unknown|2020-03-10

EPI_ISL_422581|Europe-Netherlands-unknown-unknown|2020-03-30

EPI_ISL_603344|Europe-Switzerland-Aargau-unknown|2020-09-25

EPI_ISL_427232|North_America-USA-Washington-unknown|2020-03-28

EPI_ISL_423998|Europe-United_Kingdom-England-unknown|2020-03-21

EPI_ISL_445141|North_America-USA-Louisiana-New_Orleans|2020-03-31

BetaCoV/Berlin/570067148462/2020 (101)

EPI_ISL_465087|Europe-United_Kingdom-England-unknown|2020-03-12 (7)

EPI_ISL_574884|Europe-Switzerland-Basel-Landschaft-unknown|2020-03-18

EPI_ISL_422429|Asia-Singapore-unknown-unknown|2020-02-16

EPI_ISL_510696|Europe-Switzerland-Basel-Landschaft-unknown|2020-07-10

EPI_ISL_448226|Europe-United_Kingdom-England-unknown|2020-04-18 (10)

EPI_ISL_476147|Europe-Sweden-Jamtland_Harjedalen-unknown|2020-04-09 (6)

EPI_ISL_444642|North_America-USA-New_York-Brooklyn|2020-03-30 (6)

EPI_ISL_445631|Europe-United_Kingdom-Wales-unknown|2020-04-07 (6)

EPI_ISL_483319|North_America-USA-California-San_Diego_County|2020-03-29 (5)

EPI_ISL_471467|Asia-China-Guangdong-unknown|2019-XX-XX (5)

EPI_ISL_422543|North_America-USA-New_York-Bronx|2020-03-20 (3)

EPI_ISL_453962|Europe-Portugal-unknown-unknown|2020-04-02 (2)

EPI_ISL_455323|Europe-Spain-Andalucia-unknown|2020-03-06

EPI_ISL_493364|Europe-Norway-unknown-unknown|2020-06-26

EPI_ISL_604241|North_America-USA-New_York-unknown|2020-03-16

EPI_ISL_444703|North_America-USA-New_York-Nassau_County|2020-03-30

EPI_ISL_547212|North_America-USA-Houston-unknown|2020-07-04

EPI_ISL_498584|Asia-Singapore-unknown-unknown|2020-07-04

EPI_ISL_418183|Europe-Hungary-Baranya-unknown|2020-03-17 (8)

EPI_ISL_589379|Europe-United_Kingdom-England-unknown|2020-05-06 (2)

EPI_ISL_456616|Oceania-Australia-Victoria-unknown|2020-05-19 (4)

EPI_ISL_430521|Oceania-Australia-Victoria-unknown|2020-03-23

EPI_ISL_569642|North_America-USA-New_Mexico-unknown|2020-09-03 (6)

EPI_ISL_424438|Europe-Iceland-Reykjavik-unknown|2020-03-19 (208)

EPI_ISL_449224|Europe-United_Kingdom-Scotland-unknown|2020-04-15 (7)

EPI_ISL_426963|Oceania-Australia-Victoria-unknown|2020-03-27 (7)

EPI_ISL_456441|Oceania-Australia-Victoria-unknown|2020-04-17

EPI_ISL_522688|Oceania-Australia-Tasmania-unknown|2020-05-08 (2)

EPI_ISL_509150|North_America-USA-Oregon-Multnomah_County|2020-07-10 (29)

EPI_ISL_549217|North_America-USA-Florida-unknown|2020-09-03 (4)

EPI_ISL_558602|Europe-United_Kingdom-England-unknown|2020-06-19

EPI_ISL_435072|Asia-India-Delhi-unknown|2020-03-26

EPI_ISL_458609|Europe-United_Kingdom-England-unknown|2020-03-29 (11)

EPI_ISL_511406|Europe-Portugal-unknown-unknown|2020-03-27 (7)

EPI_ISL_513760|North_America-USA-California-Orange_County|2020-05-28 (3)

EPI_ISL_516598|Europe-Switzerland-Bern-unknown|2020-08-05 (2)

EPI_ISL_525856|North_America-USA-Oregon-Washington_County|2020-06-13

EPI_ISL_589347|Europe-United_Kingdom-England-unknown|2020-05-06

EPI_ISL_587839|Europe-United_Kingdom-England-unknown|2020-10-02

EPI_ISL_533297|Europe-United_Kingdom-England-unknown|2020-06-16 (13)

EPI_ISL_474501|Europe-United_Kingdom-Wales-unknown|2020-04-24

EPI_ISL_568304|Europe-United_Kingdom-Scotland-unknown|2020-08-27

EPI_ISL_451905|Europe-Switzerland-Bern-unknown|2020-04-07

EPI_ISL_421301|North_America-USA-Wisconsin-Dane_County|2020-03-19 (11)

EPI_ISL_598533|Europe-United_Kingdom-England-unknown|2020-10-06

EPI_ISL_451867|Europe-Switzerland-Aargau-unknown|2020-04-07 (3)

EPI_ISL_491249|Europe-Portugal-unknown-unknown|2020-06-08 (3)

EPI_ISL_414527|Asia-Hong_Kong-unknown-unknown|2020-02-09 (12)

EPI_ISL_555854|Europe-United_Kingdom-England-unknown|2020-06-15

EPI_ISL_451826|Europe-Switzerland-Bern-unknown|2020-03-31

BetaCoV/Berlin/ChVir1959/2020 (147)

EPI_ISL_445769|Europe-United_Kingdom-Wales-unknown|2020-04-10 (4)

EPI_ISL_476957|Europe-Belgium-Haasrode-unknown|2020-04-11 (2)

EPI_ISL_497058|North_America-USA-Washington-Yakima_County|2020-06-15 (5)

EPI_ISL_547732|North_America-USA-Wisconsin-La_Crosse_County|2020-08-24

EPI_ISL_580641|Europe-United_Kingdom-England-unknown|2020-09-23

EPI_ISL_560587|Europe-France-Grand-Est-Soultzeren|2020-05-26

EPI_ISL_428331|North_America-USA-Wisconsin-Milwaukee_County|2020-03-28 (3)

EPI_ISL_510396|Europe-Spain-Basque_Country-San_Sebastian|2020-04-08 (3)

EPI_ISL_596327|Europe-Russia-Moscow-unknown|2020-05-30

EPI_ISL_413520|Asia-China-Beijing-unknown|2020-01-28

EPI_ISL_543804|North_America-USA-Houston-unknown|2020-06-08 (4)

EPI_ISL_423970|Europe-United_Kingdom-England-unknown|2020-03-20

EPI_ISL_461477|North_America-USA-Washington-unknown|2020-05-29

EPI_ISL_415497|Europe-Netherlands-unknown-unknown|2020-03-09 (4)

EPI_ISL_436355|Europe-Spain-Comunitat_Valenciana-Valencia_/_Sagunto|2020-03-26 (4)

EPI_ISL_576167|North_America-USA-Delaware-New_Castle_County|2020-03-25

EPI_ISL_468055|Africa-Egypt-unknown-unknown|2020-05-02

EPI_ISL_530147|North_America-USA-Washington-King_County|2020-08-13

EPI_ISL_576429|North_America-USA-Washington-unknown|2020-08-07

EPI_ISL_523096|Europe-Netherlands-Noord_Brabant-unknown|2020-05-06 (7)

EPI_ISL_417244|Europe-United_Kingdom-England-unknown|2020-03-03 (4)

EPI_ISL_603549|Europe-Switzerland-Basel-Landschaft-unknown|2020-10-08

EPI_ISL_545839|North_America-USA-Houston-unknown|2020-07-01

EPI_ISL_495283|Asia-India-Telangana-Hyderabad|2020-05-17

EPI_ISL_468298|Europe-Switzerland-Bern-unknown|2020-05-01

EPI_ISL_531910|Europe-United_Kingdom-England-unknown|2020-08-04

EPI_ISL_436131|Oceania-Australia-Victoria-unknown|2020-04-14

EPI_ISL_596530|Asia-Palestine-Jerusalem-unknown|2020-08-17 (2)

EPI_ISL_602432|Europe-Russia-Khakassia-Abakan|2020-09-25

EPI_ISL_587312|Europe-United_Kingdom-England-unknown|2020-10-01 (5)

EPI_ISL_451742|Europe-Switzerland-Basel-Landschaft-unknown|2020-03-25

EPI_ISL_602328|North_America-USA-Puerto_Rico-Ponce|2020-08-01

EPI_ISL_451811|Europe-Switzerland-Bern-unknown|2020-03-31

EPI_ISL_602955|North_America-USA-Utah-unknown|2020-06-24

EPI_ISL_426848|Oceania-Australia-Victoria-unknown|2020-03-22

EPI_ISL_589305|Europe-United_Kingdom-England-unknown|2020-05-03 (2)

EPI_ISL_559541|Europe-United_Kingdom-England-unknown|2020-05-20

EPI_ISL_501203|Asia-Malaysia-Kuala_Lumpur-unknown|2020-03-30

EPI_ISL_598485|Europe-United_Kingdom-England-unknown|2020-10-06

EPI_ISL_547726|North_America-USA-Iowa-Fayette_County|2020-08-21

EPI_ISL_454595|Europe-Croatia-Pula-unknown|2020-03-09

EPI_ISL_603280|Europe-Switzerland-Vaud-unknown|2020-09-30
